# Phase I/II study of induction chemotherapy using carboplatin plus irinotecan and sequential thoracic radiotherapy (TRT) for elderly patients with limited-disease small-cell lung cancer (LD-SCLC): TORG 0604

**DOI:** 10.1186/s12885-017-3353-y

**Published:** 2017-05-26

**Authors:** Yuki Misumi, Hiroaki Okamoto, Jiichiro Sasaki, Noriyuki Masuda, Mari Ishii, Tsuneo Shimokawa, Yukio Hosomi, Yusuke Okuma, Makoto Nagamata, Takashi Ogura, Terufumi Kato, Masafumi Sata, Sakiko Otani, Akira Takakura, Koichi Minato, Yosuke Miura, Takuma Yokoyama, Saori Takata, Katsuhiko Naoki, Koshiro Watanabe

**Affiliations:** 1 Department of Respiratory Medicine, Yokohama Municipal Citizen’s Hospital, 56 Okazawa-cho, Hodogaya-ku, Yokohama, Kanagawa Japan; 2 Department of Respiratory Medicine, Kitasato University School of Medicine, 1-15-1 Minami-ku, Sagamihara, Kanagawa Japan; 3 Department of Thoracic Oncology and Respiratory Medicine, Tokyo Metropolitan Cancer and Infectious Diseases Center Komagome Hospital, 3-18-22 Honkomagome, Bunkyo-ku, Tokyo, Japan; 4 Department of Respiratory Medicine, Kanagawa Cardiovascular and Respiratory Center, 6-16-1 Tomiokahigashi, Kanazawa-ku, Yokohama, Kanagawa Japan; 5 Department of Respiratory Medicine, Gunma Prefectural Cancer Center, 617-1 Takahayashinishi-cho, Ohta, Gunma Japan; 6 Department of Respiratory Medicine, Kyorin University School of Medicine, Kyorin University Hospital, 6-20-2 Shinkawa, Mitaka, Tokyo, Japan; 7 Division of Pulmonary Medicine, Keio University School of Medicine, Keio University Hospital, 35 Shinanomachi, Shinjuku-ku, Tokyo, Japan

**Keywords:** LD-SCLC, Irinotecan, Sequential radiotherapy, Elderly, Carboplatin, Phase I, Phase II

## Abstract

**Background:**

The role of irinotecan for elderly patients with LD-SCLC has been unclear, and the timing of TRT combined with chemotherapy has not been fully evaluated.

**Methods:**

Patients aged > 70 years with untreated, measurable, LD-SCLC, performance status (PS) 0–2, and adequate organ function were eligible. Treatment consisted of induction with carboplatin on day 1 and irinotecan on days 1 and 8, every 21 days for 4 cycles, and sequential TRT (54Gy in 27 fractions). Carboplatin doses were based on AUC of 4 and 5 (levels 1 and 2, respectively), with a fixed irinotecan dose (50 mg/m^2^). Primary objective of the phase II study was overall responce rate.

**Results:**

Forty-three patients were enrolled and forty-one were finally analyzed (median age: 75 years [range 70–86 years); males 31; PS 0/1/2, *n* = 22/18/1]. Two patients were excluded because of protocol violation (ascertained to be extensive disease). Twelve patients were accrued at phase I and the number of patients with carboplatin dose-limiting toxicities at levels-1 (*n* = 6) and −2 (*n* = 6) were 1(grade 3 hypertension) and 2 (grade 4 thrombocytopenia), respectively. The phase II trial was expanded to 29 additional patients receiving the level 1 carboplatin dose, total of 35 patients. The median number of chemotherapy cycles was 4 (range 1–4), and the median radiation dose was 54Gy (range 36–60). Toxicities were generally mild. There were 4 complete and 27 partial responses (response rate 88.6%). With a median follow-up of 52 months, the median progression-free and overall survival times of phase II were 11.2 and 27.1 months, respectively.

**Conclusions:**

Induction chemotherapy of carboplatin plus irinotecan and sequential TRT was well tolerated and effective for elderly patients with LD-SCLC. Additional confirmatory studies are warranted.

**Trial registration:**

Trial registration number: UMIN000007352

Name of registry: UMIN.

Date of registration: 1/Dec/2006.

Date of enrolment of the first participant to the trial: 6/Feb/2007.

Clinical trial registration date: 1/Feb/2006 (prospective).

**Electronic supplementary material:**

The online version of this article (doi:10.1186/s12885-017-3353-y) contains supplementary material, which is available to authorized users.

## Background

Approximately 30% to 40% of patients with small-cell lung cancer (SCLC) are older than 70 years, and in Japan, the proportion of SCLC patients who are elderly is increasing [1–3]. However, because this population of elderly patients is frequently excluded from clinical trials, there is no established standard chemotherapeutic regimen for elderly patients with SCLC. To the best of our knowledge, there have not been any randomized control trials for elderly patients with LD-SCLC, and we could only find several small phase II studies that enrolled these patients [4–7]. Concurrent chemoradiotherapy, which is standard for younger patients, might be effective; but because of the risk of a higher degree of toxicity for even “extremely healthy elderly patients”, we supposed that induction chemotherapy plus sequential radiotherapy would be more suitable for most elderly patients.

The Japan Clinical Oncology Group (JCOG) conducted a randomized control trial comparing cisplatin plus irinotecan (IP regimen) with cisplatin plus etoposide (EP regimen) for extensive disease (ED) - SCLC patients aged ≤70 years [8]. The trial was terminated at the interim analysis because IP provided significantly better overall survival (OS) than EP. However, subsequent trials [9–13] did not confirm that IP improved survival over EP. Nevertheless, the standard regimen was changed in Japan to IP for patients with ED-SCLC who were aged ≤70 years. These results suggested that irinotecan-based chemotherapy should be reasonable for elderly Japanese patients with SCLC.

Since cisplatin-based chemotherapy might be harmful for elderly patients with SCLC and comorbidities, carboplatin might be an appropriate alternative option. Rossi et al. reported a meta-analysis that showed that cisplatin and carboplatin for SCLC had different toxicity profiles, and the difference between the efficacy of the 2 agents was not statistically significant [13]. Therefore, the use of a carboplatin-based regimen for elderly patients with SCLC might be also reasonable.

According to meta-analyses [14, 15], concurrent chemoradiotherapy is more effective for patients with LD-SCLC than induction chemotherapy and sequential radiotherapy. However, some studies have found that the use of irinotecan for concurrent chemoradiotherapy led to unacceptable toxicities [16, 17]. To avoid the severe toxicity induced by thoracic radiation, a protocol consisting of carboplatin plus irinotecan induction therapy and sequential radiotherapy may be worth considering, because it addresses both safety and efficacy. In this phase I/II clinical study, we evaluated the efficacy of irinotecan for elderly LD-SCLC patients, as well as investigated its potential for a future phase III study.

## Methods

### Patient eligibility

Patients were registered at the central data center where the following eligibility criteria were confirmed: cytologically or histologically confirmed SCLC; age 70 years or older; LD, defined as disease confined to a single hemithorax (including ipsilateral and contralateral supraclavicular nodes and ≤1-cm of ipsilateral pleural effusion as measured by computed tomography (CT) without malignant cells); no prior chemotherapy or radiotherapy for SCLC; Eastern Cooperative Oncology Group (ECOG) performance status (PS) of 0–2; at least 1 measurable target lesion; no prior history of systemic chemotherapy for another cancer.

The criteria for adequate organ function included: white blood cell (WBC) count ≥4000/μL, neutrophil count ≥2000/μL, platelet count ≥100,000/μL, hemoglobin level ≥ 9.0 g/dL, serum aspirate aminotransferase (AST) and alanine aminotransferase (ALT) concentrations ≤2.0× upper limit of normal (ULN), creatinine level ≤ 1.5 mg/dL, creatinine clearance ≥40 mL/min, and arterial oxygen pressure ≥ 70 Torr.

Patients were excluded from the study if they had either interstitial pneumonia or pulmonary fibrosis on chest radiography, or any severe concomitant disease (severe cardiac disease, severe infection, uncontrolled diabetes mellitus, severe hepatic disorder, active bleeding). Written informed consent was obtained from every patient. The protocol was approved by the institutional review committee of each of the participating institutions.

### Evaluation for enrollment

All patients were required to undergo CT of the thorax and the upper abdomen, either CT or magnetic resonance imaging (MRI) of the brain, and either a radioisotopic bone scan or positron emission tomography (PET) for assessing disease stage. A complete blood cell count and a blood chemistry panel were also obtained at enrollment. After the treatment protocol was started, chest radiography was performed at least 1 time per chemotherapy cycle, and blood testing was performed every week. CT was repeated every 2 cycles to evaluate the target lesions. Tumor response was assessed using the Response Evaluation Criteria in Solid Tumors version 1.0, and toxicity was assessed using the National Cancer Institute Common Terminology Criteria for Adverse Events, version 3.0.

### Phase I section

The primary endpoint for the phase I trial was to determine the recommended dose (RD). Based on a previous study [14], the following dose levels of irinotecan were evaluated: level 1, 50 mg/m^2^ of irinotecan intravenously (IV) on days 1 and 8 plus carboplatin IV with a dose based on the area under the curve (AUC) of 4 on day 1; level 2, 50 mg/m^2^ of irinotecan IV on days 1 and 8 plus carboplatin AUC 5 on day 1. Irinotecan was not administered on day 8 for WBC < 3000/mm^3^, platelet count <100,000/mm^3^, or if diarrhea of grade 1 or higher occurred. When the toxicities did not recover until 3 days ahead of planned day 8, the day 8 irinotecan administration was withdrawn.

Chemotherapy was repeated for up to 4 cycles, unless disease progression was observed or there was unacceptable toxicity. However, termination of the chemotherapy protocol and initiation of radiotherapy was permitted if the response after the second chemotherapy cycle was stable disease. A treatment delay of up to 2 weeks was permitted. Granulocyte colony-stimulating growth factor (G-CSF) could be used in accordance with the package insert. If G-CSF was administered, the criteria for administering the next chemotherapy cycle should be satisfied both after day 21 and 2 or more days after the discontinuation of G-CSF.

Antiemetic prophylaxis with 5-HT_3_ receptor antagonists plus dexamethasone was routinely used. Dose modification was only allowed for the level 2 cohort of patients, as follows: grade 4 leukopenia or neutropenia lasting 4 days or more; grade 4 thrombocytopenia; or grade 3 non-hematological toxicities, except for nausea/vomiting, anorexia, hyponatremia, and creatinine elevation. When dose modification was needed, the next treatment cycle was started with carboplatin AUC 4 on day 1 plus irinotecan 40 mg/m^2^ on days 1 and 8 every 21 days. When level 1 patients developed toxicity to these modified doses, the chemotherapy protocol was terminated. Likewise, when level 2 patients developed similar toxicity again after dose modification, the chemotherapy protocol was terminated.

**Fig. 1:**
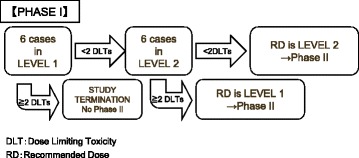
Dose escalation schematic of Phase I

The dose level was escalated based on the development of toxicity during chemotherapy cycles and was not escalated for each patient. Dose limiting toxicity (DLT) was considered to be any of the following adverse events observed during the initial 2 chemotherapy cycles: grade 4 thrombocytopenia; grade 4 febrile neutropenia; grade 4 neutropenia or leucopenia for ≥4 days; grade 3 nonhematological toxicity (except for nausea/vomiting, hyponatremia, and creatinine elevation); and delay of the next cycle for ≥14 days. The dose escalation schematic is shown in Fig. 1; if 1 or zero of the initial 6 patients receiving level 1 chemotherapy developed DLT, then 6 patients received level 2 treatment. If 1 or zero of the 6 patients receiving level 2 chemotherapy developed DLT, the dose was considered to be the RD. If 2 or more of the patients receiving level 2 chemotherapy developed DLT, level 1 was considered to be the RD. If 2 or more of the initial 6 patients receiving level 1 chemotherapy developed DLT, the RD could not be defined, and no phase II trial would be conducted. DLT was monitored until the end of the first 2 chemotherapy cycles.

### Phase II section and statistical analysis

The primary endpoint of the phase II study was the overall response rate (ORR). Based on the Simon two-stage design, the phase II trial was designed to detect the difference between ORRs of 0.60 and 0.80 with more than 80% power (exact binomial test for one sample proportion, 1-sided ˛ = 0.05). Thirteen patients, including those who received the RD in the phase I trial, were enrolled in an interim analysis, and the new regimen was considered worthy of further investigation if tumor response was observed in ≥9 patients. For the phase II study, an additional 22 patients were enrolled; and the total number of patients in the phase II trial was 35. The secondary endpoints were OS, Progression-free survival (PFS), toxicity, and rate of treatment completion. The patient cohort completing treatment was considered to be those patients who received both 2 cycles of protocol chemotherapy and ≥50 Gy of thoracic radiotherapy (TRT). The Kaplan–Meier method was used to estimate the median values of time-to-events, such as OS and PFS; and the confidence intervals (CIs) were calculated using the Brookmeyer and Crowley method. All statistical analyses were performed using BellCurve for Excel (Social Survey Research Information, Tokyo, Japan).

### Thoracic radiotherapy

TRT was begun on day 22 of the fourth chemotherapy cycle and was administered at 2Gy/day for 5 consecutive days/week for a total of 54 Gy. Postchemotherapy treatment volumes were used for radiotherapy. Every patient underwent three-dimensional conformal radiation therapy (3D–CRT) planning. The dose constraints for the lung were a mean lung dose (MLD) <20 Gy and a V20 of 35% or less. The target volume included the lung tumor and involved lymph nodes, with margins of 1.0–1.5 cm. The maximum dose to the spinal cord was 40 Gy.

To guarantee the intensity of radiotherapy, the total duration of TRT was ≤56 days. Thoracic radiotherapy alone without the use of chemotherapy was permitted on day 22 of the second chemotherapy cycle if tumor response was not obtained. The initiation of TRT was permitted only for patients with the following clinical parameters: WBC ≥ 2000/mm^3^, PaO_2_ ≥ 65 Torr on room air, PS = 0–2, and no interstitial pneumonia or pulmonary fibrosis on chest radiography. TRT was suspended when the patient developed 1 or more of the following: grade 3 nonhematological toxicities: PS of 3–4, grade ≥ 2 pneumonitis, temperature ≥ 38 °C, or grade ≥ 2 hypoxemia with PaO2 decrease of 10 Torr. Thoracic radiotherapy was restarted if there was improvement, but antifebrile agents within 24 h of TRT were not allowed. The TRT protocol was terminated for grade ≥ 3 pneumonitis or if TRT had been suspended for 14 days because of the other toxicities.

### Treatment after protocol

Antitumor treatment was permitted after the protocol when the tumor was confirmed to be progressive disease. Patients who achieved a complete response (CR) could receive prophylactic cranial irradiation (PCI), but PCI was not mandatory.

## Results

Forty-three patients were enrolled from December 2006 through June 2013 at 12 institutions. Thirty-seven patients, which included 6 patients from the phase I trial who were treated with the RD level, were enrolled in the phase II trial (2 patients were found to have ED-SCLC after registration and were excluded from the analysis). The median age of all eligible patients in the study was 75 years (range, 70–86 years). Only one patient had an ECOG PS of 2; 10 patients had N3 disease (Table 1).

**Table 1:** Baseline characteristics

	Phase I		Phase II	
Number of patients	12		35*	
Age (years)				
median	72		75	
range	70–81		70–86	
Sex				
male	9	75%	25	71%
female	3	25%	10	29%
ECOG PS				
0/1/2	8/4/0		17/17/1	
TNM factors				
T 1/2/3/4	2/6/0/4		10/14/5/6	
N 0/1/2/3	0/4/5/3		2/8/17/8	
Brinkman’s index				
median	1000		1000	
range	0–3600		0–2950	

*ECOG PS* Eastern Cooperative Oncology Group Performance Status

Asterisk: including 6 patients who recieved level 1 treatment at phase I portion

### Phase I MTD and DLT

The phase I trial included 12 patients (Table 2). At level 1, 1 of 6 patients developed DLT (grade 3 hypertension). The dose was then escalated to level 2, where 6 patients were enrolled and treated. At level 2, 2 of 6 patients developed a DLT of grade 4 thrombocytopenia. Moreover, 1 patient with grade 4 thrombocytopenia also developed grade 4 neutropenia and grade 3 glaucoma. Therefore, level 1 was considered to be the RD.

**Table 2:** Worst grade of adverse events observed during chemotherapy at phase I dose

	LEVEL 1 *n* = 6			LEVEL 2 *n* = 6		
	Grade (NCI-CTC ver. 3.0)					
Toxicity	1–2	3	4	1–2	3	4
Leukocytes	5	1	0	5	1	0
Neutrophils	4	2	0	3	2	1
Hemoglobin	5	1	0	5	1	0
Platelets	6	0	0	4	0	2*
Fatigue	6	0	0	6	0	0
Anorexia	6	0	0	6	0	0
Nausea	6	0	0	6	0	0
Vomiting	6	0	0	6	0	0
Esophagitis	0	0	0	0	0	0
Diarrhea	6	0	0	6	0	0
Constipation	6	0	0	6	0	0
Alopecia	6	0	0	6	0	0
FN	6	0	0	6	0	0
Pheumonitis	6	0	0	6	0	0
Hypertension	5	1*	0	6	0	0
Glaucoma	6	0	0	5	1*	0

*NCI-CTC* National Cancer Institute - Common Toxicity Criteria

*FN* Febrile Neutropenia

Asterisk: dose limiting toxicity

### Phase II tumor response

Among the 35 patients treated with the RD (level 1), 4 achieved CR and 27 achieved partial response (PR). Therefore, the ORR was 88.6% (95% CI, 73.3%–96.8%), and the null hypothesis for the phase II trial was accepted. Two patients had stable disease, and none had progressive disease (PD). Two patients who terminated protocol treatment because of grade 3 pneumonitis during TRT were categorized not evaluable for response. In addition, these 2 patients received second-line treatment before disease progression was confirmed. The disease control rate was 94.3% (95% CI, 80.8%–99.3%).

### Toxicity of chemotherapy during the phase II trial and treatment cycles

The toxicities that occurred during treatment of the 35 patients at the RD level are shown in Table 3. Although grade 3 or higher neutropenia and thrombocytopenia were observed in 25.7% and 2.8% of the patients, respectively, there were no treatment-related deaths, and all the patients with grade ≥ 3 toxicities recovered. The only grade 4 nonhematological toxicity was hyponatremia. Only grade 2 or lower diarrhea occurred.

**Table 3:** Worst grade of adverse events observed during chemotherapy and TRT at phase II

	During phase II chemotherapy				During TRT			
*n* = 35	Grade (NCI-CTC ver. 2.0)							
Toxicity	1–2	3	4	3+4 (%)	1–2	3	4	3+4 (%)
Leukocytes	27	3	2	14.3	14	0	0	0
Neutrophils	13	14	4	51	10	3	0	8.4
Hemoglobin	20	11	2	37.1	21	3	0	8.4
Platelets	24	2	2	11.4	4	1	0	2.8
Hyponatremia	1	2	1	8.6	1	1	0	2.8
AST/ALT	12	1	0	2.8	5	0	0	0
Fatigue	18	0	0	0	6	0	0	0
Anorexia	21	5	0	14.3	6	0	0	0
Nausea/Vomiting	21	1	0	2.8	2	0	0	0
Skin rash	2	0	0	0	5	0	0	0
Esophagitis	0	0	0	0	12	0	0	0
Diarrhea	18	0	0	0	3	0	0	0
Stomatitis	0	1	0	2.8	3	1	0	2.8
Alopecia	14	0	0	0	10	0	0	0
Infection	1	0	0	0	0	0	0	0
FN		2	0	5.7		0	0	0
Pheumonitis	0	1	0	5.7	4	2	0	5.7

*TRT*Thoracic Radiotherapy

*NCI-CTC* National Cancer Institute - Common Toxicity Criteria

*AST* Aspartate transaminase, *ALT* Alanine transaminase, *FN* Febrile Neutropenia

G-CSF was administered to 19 patients (54%), and G-CSF was administered during more than 1 chemotherapy cycle to 15 of 19 patients.

No dose reduction was allowed at the RD. Seven patients terminated the chemotherapy protocol because of the following toxicities: prolonged thrombocytopenia, grade 3 hypertension, grade 3 pneumonia, grade 3 ALT elevation, grade 3 febrile neutropenia, grade 3 creatine kinase elevation, or prolonged neutropenia. Of 35 patients treated at the RD level, 28 (80%) completed 4 cycles.

### TRT dose and TRT toxicity during the phase II trial

The TRT doses ranged from 36 to 60 Gy, and 25 patients (71.4%) received the planned dose of 54 Gy. One patient terminated TRT because of disease progression. The toxicities that occurred during TRT are summarized in Table 3. Grade 3 radiation pneumonitis was observed in 2 (5.7%) patients, one receiving a total TRT dose of 44 Gy and the other receiving 54 Gy. The treatment was terminated, and each patient was treated with systemic corticosteroids. Both patients achieved complete recovery; no tumor progression had been detected by the time of last follow up.

### Treatment completion

Of the 35 patients in the phase II study, 29 (82.9%) received 2 or more cycles of protocol chemotherapy and TRT ≥50 Gy.

### Follow up after the phase II study

Seven (20%) of 35 patients underwent PCI. Twenty-three patients (66%) developed recurrence, and 18 patients received other systemic chemotherapy. Five patients (14%) underwent brain radiotherapy. The first sites of recurrence were primarily distant metastases (central nervous system: *n* = 11; others: *n* = 12), and no obvious tendency was observed. Eleven patients developed locoregional recurrence in the TRT field, and 4 of these also developed distant metastasis. Two patients died of another disease without confirmation of relapse, and the others died of SCLC progression.

### Progression-free and overall survival

**Fig. 2:**
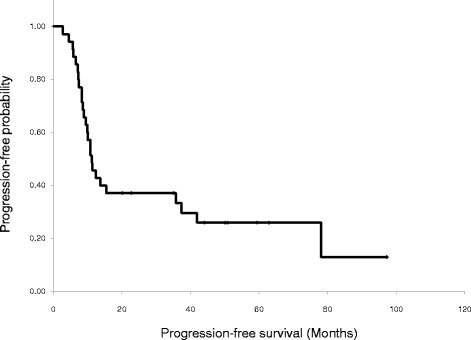
Progression-free survival

**Fig. 3:**
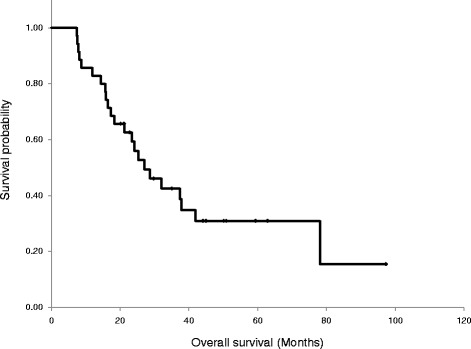
Overall survival

The median PFS of all 41 patients was 10.8 months (95% CI, 9.3–12.3 months). The median PFS of the phase II trial was 11.2 months (95% CI, 8.5–13.8, months; Fig. 2). The median OS of all 41 patients was 25.3 months (95% CI, 18.0–32.6 months). The median OS of the phase II trial was 27.1 months (95% CI, 17.0–37.2 months; Fig. 3). The median duration of follow up of patients in the phase II trial was 52 months.

## Discussion

Based on several reports, the standard treatment for patients with LD-SCLC is concurrent chemoradiotherapy, regardless of the age of the patient [18]. However, concurrent chemotherapy is sometimes too toxic for fragile elderly patients, even if they are otherwise suitable for antitumor treatment. Individualized treatment for patients with SCLC has not yet been developed. Therefore, a treatment regimen that is applicable for most elderly patients is needed, especially in countries that have an aging population, such as Japan.

Some studies have found that irinotecan is effective for elderly patients with SCLC. We recently reported the results of 2 clinical trials that examined the efficacy and toxicity of CI regimen for SCLC, and found that the CI regimen appeared to have promise for the treatment of SCLC [19, 20]. Moreover, other investigators also found that CI regimens showed acceptable activity and toxicity for patients with SCLC, not only for elderly but also younger patients [21–23]. Schmittel et al. conducted a randomized phase III trial to compare CI with carboplatin plus etoposide (CE regimen) for the treatment of patients with ED-SCLC [21]. Although this trial failed to show the superiority of irinotecan over etoposide in combination with carboplatin with regard to PFS as the primary endpoint, OS was marginally better with the CI regimen. Another study that compared CI with CE for ED-SCLC was conducted in Norway [22]. The primary endpoint of OS was significantly better in the CI arm, which also obtained slightly better quality of life (QOL).

We conducted a safety and efficacy phase I/II study of the CI regimen and sequential TRT. The results were generally acceptable, and not inferior to previous studies of elderly patients [24, 25]. Several long-term survivors were observed despite the increased mean age of the study cohort. The estimated 5-year survival rate of 30% was very promising (Table 4). However, our study required a long enrollment period because there were few patients with LD-SCLC who had adequate organ function for the treatment used in our study.

**Table 4:** Comparison between our study and the other LD-SCLC studies

Author	Age	Regimen	TRT	Survival
Turissi et al. [24]	all	CDDP plus ETP	45Gy, once/ twice a daily, concurrent	MST:19 months (once) MST:23 months (twice)
Jeremic et al.	≥70	CBDCA plus ETP	45Gy, twice a daily, concurrent	2-year survival:32% 5-year survival:13%
Okamoto et al. [25]	≥70	CDDP plus ETP	45Gy, twice a daily, concurrent	MST:24.1 months
This study	≥70	CBDCA plus IRINOTECAN	54Gy, once a daily, sequential	MST:27.1 months

*CDDP* Cisplatin

*ETP* Etoposide

*MST* Median Survival Time

*TRT* Thoracic Radiotherapy

The toxicities in our study were generally mild. All the patients with grade-3 or higher adverse events generally improved, and there were no treatment-related deaths.

Most patients with SCLC have a history of cumulative smoking exposure, which leads to increased frailty [26, 27]. Therefore, the recruitment of elderly SCLC patients for chemoradiotherapy is generally difficult. Nevertheless, our study protocol achieved good efficacy and acceptable toxicity. In addition, the survival results were not inferior to the results from a recent study of younger patients with LD-SCLC [28, 29]. Although some patients had to terminate chemotherapy, all of the study patients achieved sufficient recovery from adverse effects, so that they could undergo sequential TRT.

Irinotecan sometimes causes severe diarrhea or interstitial pneumonitis in patients with SCLC. However, our study patients with diarrhea generally had a mild and manageable course, which might be attributed to the use of a dose of irinotecan that was lower than the standard dose for SCLC.

According to the tolerability data from the phase II study, we believe that treatment using the level 2 dose would not be suitable for RD. We previously administered the same chemotherapy (level 1 of this protocol) to elderly ED-SCLC patients, and 7 patients (70%) required treatment delays of ≥7 days because of grade 3 neutropenia or grade 3 thrombocytopenia [20]. Although the patients had ED-SCLC, the other eligibility criteria of their study were similar to those in our study.

Although the standard therapy for LD-SCLC is “concurrent chemoradiotherapy”, we used sequential TRT in this study. Several meta-analyses [15, 30–32] reported that early initiation of TRT was advantageous, but we were concerned that concurrent radiotherapy would lead to severe toxicities in this study cohort of frail patients. Syukuya et al. reported that they selected only 5 of 20 elderly (≥75 years) patients with LD-SCLC to receive concurrent TRT [33]. Elderly patients with LD-SCLC tend to have a history of heavy smoking and many comorbidities, so their decision to only enroll 5 patients for concurrent chemo radiotherapy was based on the frailty of their patients. Moreover, 2 of their 5 elderly patients (40%) who received concurrent TRT terminated treatment because of severe toxicity. The results of their study indicated that the safety of treatments for most elderly patients with LD-SCLC is of particular concern. Okamoto et al. reported that the first cycle of the EP regimen plus concurrent TRT for elderly patients with LD-SCLC led to a high frequency of febrile neutropenia (8 of 12) [19]. We do not rule out the use of concurrent TRT, but we would not generally consider using it for elderly LD-SCLC patients.

In our study, the dose intensity of TRT was generally satisfactory, and few patients developed severe pneumonitis and esophagitis. The sequential TRT field was usually restricted because of tumor shrinkage due to induction chemotherapy. The smaller field might have accounted for the reduced rate in our patients of severe toxicities due to TRT.

The Japanese JCOG 0202 study was published while our study was ongoing [29]. The investigators compared IP with EP, using concurrent TRT, for patients with LD-SCLC aged 20–70 years. They anticipated that the IP regimen would be superior, but the OS (primary endpoint) of patients receiving IP was not improved over EP. The results of their study cast doubt on the superiority of irinotecan over etoposide; however, results of another study have supported the superiority of irinotecan [34]. Additional trials that compare these 2 agents are needed.

There are several limitations to this study. It was not a comparative study, had a small phase II component, and no results allowed us to make a conclusion regarding the superiority of the CI regimen. The radiation fields for sequential TRT might have accounted for the lower incidence of TRT toxicity; whether or not sequential TRT might have been the best strategy for managing the disease is unclear. In addition, because the toxicities in phase II were generally mild, an RD midway between levels 1 and 2 might be better for phase II. Although this study allowed the inclusion of patients with a PS of 2, only 1 such patient was enrolled. Therefore, we could not clearly show the efficacy and safety for patients with a PS of 2. Moreover, we enrolled 43 patients from 12 institutions, all of whom had an ECOG PS of 0–2. Considering the very small number of average patients per institute, there seems to have been great heterogeneity based on institutions, and it might be difficult to popularize in general. Finally, we could not easily determine if the patients included in our study could safely receive cisplatin based regimens. A meta-analysis found that there was no significant difference between the efficacy of cisplatin and of carboplatin for the treatment of SCLC [13], so we considered it was not suboptimal treatment.

## Conclusions

Induction chemotherapy consisting of a CI regimen and sequential TRT was well tolerated and effective for elderly patients with LD-SCLC. Further confirmatory studies are warranted.

## Additional file

Additional file 1: The list of the ethics committees (IRBs) which approved this study. (DOCX 13 kb)

## Abbreviations

3D–CRT: Three-dimensional conformal radiation therapy
ALT: A lanine aminotransferase
AST: Aspartate aminotransferase
AUC: Area under the curve
Cis: Confidence intervals
CR: Complete response
CT: Computed tomography
DLT: Dose-limiting toxicity
ECOG: Eastern Cooperative Oncology Group
ED: Extensive disease
EP: Cisplatin plus Etopside
G-CSF: Granulocyte colony-stimulating factor
IP: Cisplatin plus Irinotecan
JCOG: The Japan Clinical Oncology Group
LD: Limited disease
MLD: Mean lung dose
MRI: Magnetic resonance imaging
ORR: Overall response rate
OS: Overall survival
PCI: Prophylactic cranial irradiation
PD: Progressive disease
PET: Positron emission tomography
PFS: Progression-free survival
PR: Partial response
PS: Performance status
QoL: Quality of life
RD: Recommended dose
SCLC: Small cell lung cancer
TRT: Thoracic radiotherapy
ULN: Upper limit of normal
WBC: White blood cell
