# Supplementary material for: Phase I/II study of induction chemotherapy using carboplatin plus irinotecan and sequential thoracic radiotherapy (TRT) for elderly patients with limited-disease small-cell lung cancer (LD-SCLC): TORG 0604
Source: BMC Cancer. 2017 May 26;17:377. doi: 10.1186/s12885-017-3353-y (PMC5446686; doi:10.1186/s12885-017-3353-y)
Supplement: Additional file 1: — The list of the ethics committees (IRBs) which approved this study. (DOCX 13 kb) [file 12885_2017_3353_MOESM1_ESM.docx]

This study was approved by the ethics committees (IRBs) of the institutes listed below;

Yokohama Municipal Citizen’s Hospital

Kitasato University School of Medicine

Tokyo Metropolitan Cancer and Infectious Diseases Center Komagome Hospital

Kanagawa Cardiovascular and Respiratory Center

Gunma Prefectural Cancer Center

Kyorin University Hospital

Keio University Hospital

Fujisawa City Hospital

Toranomon Hospital

Osaka Medical College Hospital

Kanagawa Cancer Center

Teikyo University Hospital
